# Pharmacodynamic Monitoring of RO5459072, a Small Molecule Inhibitor of Cathepsin S

**DOI:** 10.3389/fimmu.2017.00806

**Published:** 2017-07-17

**Authors:** Michel Theron, Darren Bentley, Sandra Nagel, Marianne Manchester, Michael Gerg, Thomas Schindler, Ana Silva, Barbara Ecabert, Priscila Teixeira, Camille Perret, Bernhard Reis

**Affiliations:** ^1^Roche Pharmaceutical Research and Early Development, Pharmaceutical Sciences, Roche Innovation Center Basel, F. Hoffmann-La Roche Ltd., Basel, Switzerland; ^2^Roche Pharmaceutical Research and Early Development, Clinical Pharmacology, Roche Innovation Center Basel, F. Hoffmann-La Roche Ltd., Basel, Switzerland; ^3^Roche Diagnostics, Roche Innovation Center Munich, F. Hoffmann-La Roche Ltd., Penzberg, Germany; ^4^Roche Pharmaceutical Research and Early Development, Immunology and Inflammation, Roche Innovation Center Basel, F. Hoffmann-La Roche Ltd., Basel, Switzerland

**Keywords:** cathepsin S, pharmacodynamic, biomarker, clinical, neoepitope, major histocompatibility complex class II, antigen presentation

## Abstract

Major histocompatibility complex class II (MHCII)-restricted antigen priming of CD4^+^ T cells is both involved in adaptive immune responses and the pathogenesis of autoimmune diseases. Degradation of invariant chain Ii, a protein that prevents premature peptide loading, is a prerequisite for nascent MHCII–peptide complex formation. A key proteolytic step in this process is mediated by cathepsin S. Inhibition of this cysteine protease is known to result in the intracellular accumulation of Lip10 in B cells. Here, we describe the development and application of a neoepitope-based flow cytometry assay measuring accumulation of Lip10. This novel method enabled the investigation of cathepsin S-dependent MHCII maturation in professional antigen-presenting cell (APC) subsets. Inhibition of cathepsin S by a specific inhibitor, RO5459072, in human PBMC *ex vivo* resulted in accumulation of Lip10 in B cells and myeloid dendritic cells, but not in plasmacytoid dendritic cells and only to a minor degree in monocytes. We qualified Lip10 as a pharmacodynamic biomarker by showing the cathepsin S inhibitor-dependent accumulation of Lip10 *in vivo* in cynomolgus monkeys treated with RO5459072. Finally, dosing of RO5459072 in a first-in-human clinical study (www.ClinicalTrials.gov, identifier NCT02295332) exhibited a dose-dependent increase in Lip10, confirming target engagement and demonstrating desired pharmacologic inhibition *in vivo*. The degree of cathepsin S antagonist-induced maximum Lip10 accumulation in APCs varied significantly between individuals both *in vitro* and *in vivo*. This finding has not been reported previously using alternative, less sensitive methods and demands further investigation as to the potential of this biomarker to predict response to treatment. These results will help guide subsequent clinical studies investigating the pharmacokinetic and pharmacodynamic relationship of cathepsin S inhibitor RO5459072 after multiple dosing.

## Introduction

Major histocompatibility complex (MHC) class II (MHCII) molecules are central to adaptive immune responses. MHCII-restricted autoantigen priming of CD4^+^ T cells is thought to play a role in the pathogenesis of a number of autoimmune diseases. CD4^+^ or helper T cells, which include the well-known Th1 and Th2 subsets as well as the more recently described Th17 and regulatory subsets, are implicated in many autoimmune diseases such as rheumatoid arthritis, multiple sclerosis, Sjögren’s syndrome, and inflammatory bowel diseases (1–3). The interaction between professional antigen-presenting cells (APCs) and CD4^+^ T cells has become a target for various treatment approaches including therapeutic modulation of the MHCII antigen presentation pathway (4–6).

**Figure 1:**
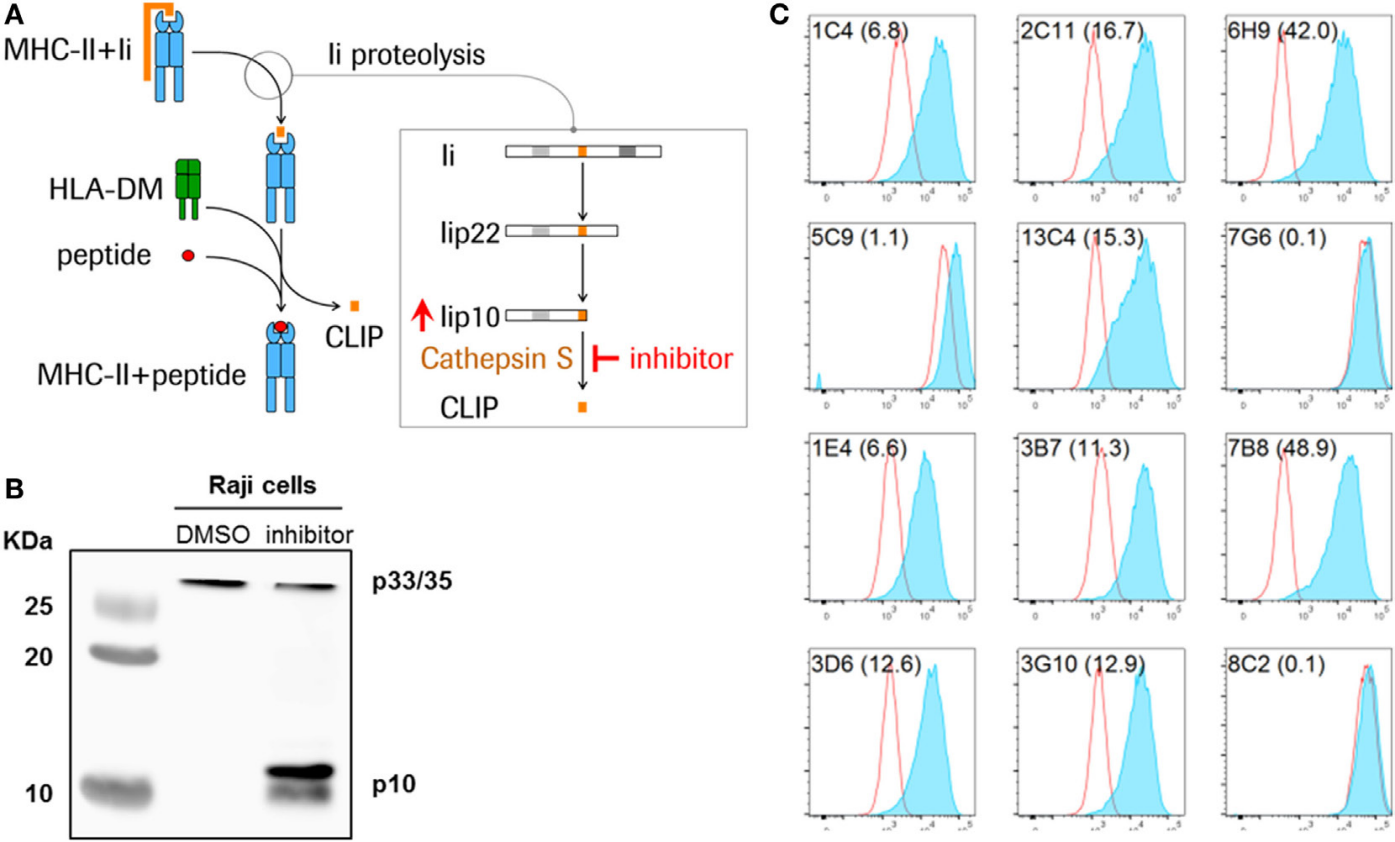
Detection of Lip10 accumulation in RAJI B cell line upon cathepsin S-specific inhibition. **(A)** Schematic diagram of invariant chain (Ii) proteolytic removal as part of the major histocompatibility complex (MHC) class II presentation pathway. Inhibition of cathepsin S activity leads to an accumulation of cathepsin S substrate Lip10 in professional antigen-presenting cells. **(B)** Lip10 accumulation after cathepsin S inhibition was detected by western blot using an anti-CD74 antibody in RAJI cells incubated for 20 h with cathepsin S-specific inhibitor RO5459072. **(C)** Twelve antibody clones raised in rabbits against the C-terminal end of Lip10 were tested for the ability to detect intracellular Lip10 accumulation by staining RAJI cells incubated for 20 h with RO5459072 in a flow cytometry-based assay. Lip10 median fluorescence intensity (MFI) is displayed for DMSO-treated (red line) and RO5459072-treated (solid blue) RAJI cells. The stain index, calculated for each clone, with (MFI of cathepsin S inhibitor-treated cells − MFI of DMSO-treated cells)/SD of DMSO-treated cells, is displayed in parenthesis after each clone name.

Newly synthesized MHCII molecules are initially associated with the CD74 invariant chain (Ii), a chaperone molecule required for the maturation of the MHCII complex by facilitating appropriate folding, preventing premature peptide loading and directing nascent MHCII molecules to the late endosomal compartment for peptide loading (7, 8). Proteolytic cleavage of Ii is required for MHCII maturation, because only then can antigenic peptides be acquired and an MHCII–peptide complex formed. The proteolysis of Ii occurs as a series of sequential steps, ultimately leaving the class II-associated invariant chain peptide (CLIP), a 24-amino-acid fragment, in the peptide-binding cleft of MHCII molecules (Figure 1A) (9, 10). The MHC-like molecule HLA-DM then facilitates the exchange of CLIP with 10–14 amino acid long peptides within the endolysosomal compartment, after which the MHCII–peptide complex is transported to the cell surface for presentation of the peptide antigen to CD4^+^ T cells.

In B cells, cathepsin S is the single enzyme responsible for the last step of the Ii proteolytic cascade, mediating the cleavage of a 10-kDa fragment of the Ii (Lip10), to form CLIP, the critical step at which the endoplasmic reticulum (ER) retention signal is removed (8, 11). Inhibition of this cysteine protease is known to result in the intracellular accumulation of Lip10 (Figures 1A,B). Inactivation of cathepsin S leads to reduced antigen presentation through multiple mechanisms. First, inhibiting cathepsin S prevents cleavage of Lip10, leading to prolonged association of MHCII with Lip10. The presence of an ER retention signal in Lip10 results in partial down-modulation of MHCII molecules on the cell surface of APC and intracellular accumulation instead. Second, the antigen binding cleft of MHCII is blocked by the delayed or absent removal of Lip10/CLIP, preventing exchange with peptide antigens in the endolysosomal compartment. Third, the absence of cathepsin S-mediated cleavage of proteins in the endolysosomal compartment can alter the pool of peptides available for loading to MHCII molecules (10).

Mice deficient in cathepsin S show impaired Ii processing in both dendritic cells and B cells, but only moderately in macrophages, and exhibit a defect in antigenic peptide presentation and antibody class switching (12, 13). In alignment with its suggested key role in antigen presentation, pharmacologic or genetic inhibition of cathepsin S in preclinical models alleviates pathogenic symptoms in various autoimmune diseases such as multiple sclerosis and arthritis (12, 14, 15). The unique and cell type-specific role of cathepsin S thus represents an opportunity for potential therapeutic intervention in autoimmune diseases with significant pathogenic involvement of CD4^+^ T cells, and therefore cathepsin S has become an appealing target for drug development (14–16). The cathepsin S inhibitor RO5459072 is the clinical lead molecule at F. Hoffman-La Roche, designed for treatment of autoimmune diseases associated with MHCII peptide loading and is a selective, potent, and competitive oral inhibitor of the active site of cathepsin S. RO5459072 has been shown to reduce CD4 T cell and dendritic cell activation as well as autoantibody production in a preclinical model of spontaneous systemic lupus erythematosus and lupus nephritis (16).

Rational and efficient development of new therapeutic molecules requires the implementation of informative biomarkers. Pharmacodynamic assays are an important tool for understanding the relationship between a molecule, its intended biological effects and dose–response relationships, and ultimately clinical benefit (17, 18). To date, therapeutic targeting of cathepsin S has focused on Lip10 measured by western blotting as an indication of target engagement and quantitative readout of drug exposure (14, 15). However, the application of western blotting in a clinical setting brings significant drawbacks, namely low dynamic range, difficulty to standardize results across a larger sample size, relative labor intensiveness, and resulting low throughput. Taken together, these constraints make western blotting a technology suboptimal for generation of reliable endpoint data for dose finding in clinical studies. To overcome the limitations of previously applied methods for monitoring pharmacologic inhibition of cathepsin S and enable use of pharmacodynamic biomarker data for decision-making in early clinical studies, we report the development and successful implementation of a pharmacodynamic biomarker assay using flow cytometry to detect intracellular Lip10 accumulation in professional APCs. This method is novel due to the employment of an antibody specific for the c-terminal neoepitope formed after upstream proteolytic cleavage of the Lip10 precursor Lip22. We show the application of this assay as a tool to investigate the requirement of cathepsin S for MHCII maturation in APC subpopulations. We provide proof-of-concept that the assay can measure pharmacodynamic activity in RO5459072-treated non-human primates. Finally, when applied to a human clinical study, the assay confirmed target engagement and enabled selection of an optimal drug dose for achieving maximal biological effects through optimized target inhibition *in vivo*.

## Materials and Methods

### Cathepsin S Antagonists and Neoepitope-Specific Antibody

RO5459072 and RG7236 are cathepsin S antagonists synthesized at F. Hoffmann-La Roche (Basel, Switzerland), acting as competitive inhibitors of the active site of cathepsin S. RO5459072 is the clinical lead molecule which was used in all experiments if not specifically otherwise mentioned. The development of these inhibitors has been described elsewhere (19). Monoclonal antibodies specific for Lip10 (Roche Diagnostics, Penzberg, Germany) were raised in rabbit following immunization with KLH-coupled peptides corresponding to the various C-terminal ends of Lip10 (neoepitope). For the immunization, five different peptides, corresponding to the potential C-terminal neoepitopes of Lip10 after protease cleavage, were synthesized. The peptides were spanning the amino acids 108–116, 108–117, 108–118, 108–119, and 108–120 of Lip10. Three rabbits per immunogen were immunized for the antibody development. Antibody development was performed using the B-cell PCR technology described elsewhere (20). To analyze the specificity of the developed clones, N-terminal biotinylated screening peptides were synthesized (108–116, 108–117, 108–118, 108–119, 108–120, and 108–121), and all clones were tested against all peptides. Additionally, fusion peptides consisting of an N-terminal epitope (12–28; DQKPVMDDQRDLISNNE) of Lip10 fused to the various C-terminal ends were synthesized for specificity testing in a sandwich ELISA format. Therefore, the fusion peptides were captured on plate surfaces *via* an antibody specific for the N-terminal epitope (PIN.1, Abcam). Twelve clones were evaluated as part of the development of a cathepsin S activity assay. The clones and their respective specificity are listed in Table S1 in Supplementary Material.

### Blood Sample Collection and PBMC Enrichment

Human blood samples from healthy volunteers were collected under the Blood Donation for Research Purposes program at F. Hoffmann-La Roche, Basel, Switzerland. Written informed consent was obtained from all donors. Experiments were conducted in accordance with the Declaration of Helsinki and all applicable regulatory and ethical requirements. Cynomolgus blood samples were drawn from adult *Macaca fascicularis* monkeys (Bioprim, Baziege, France), housed and cared for according to the Swiss Animal Welfare Act and Ordinance. The work described here was carried out in accordance with the EU directive 2010/63/EU for animal experiments. All blood samples were collected in BD Vacutainer collection tubes containing sodium heparin (BD, Allschwill, Switzerland).

PBMC were enriched from whole blood by gradient separation with either Ficoll-Paque PLUS (GE Healthcare Europe, Glattbrug, Switzerland) for human samples or a 1:9 mixture of PBS and Ficoll-Paque PLUS for cynomolgus monkey samples.

### *Ex Vivo* Treatment of Cells with Cathepsin S Inhibitor

Enriched PBMC or cultured RAJI cells (Leibniz Institute DSMZ, Braunschweig, Germany) were resuspended in RPMI 1640 with GlutaMAX-I, supplemented with 10% heat-inactivated FBS, 50µM 2-mercaptoethanol, and 100 U/mL penicillin–streptomycin, and incubated with a serial dilution of a cathepsin S inhibitor pre-titrated in DMSO. Cells were seeded in 48-well plates and incubated for 20 h at 37°C. The cells were then harvested and washed with PBS before being processed further for Lip10 detection.

### Cathepsin S Activity in Cynomolgus Monkeys Dosed with Cathepsin S Inhibitor

Blood samples from six adult cynomolgus monkeys weighing 8–12 kg were collected and PBMC enriched for Lip10 detection (time-point 0 h). The monkeys were subsequently divided into two groups and given a single oral dose of either 50 or 200 mg/kg of RO5459072, a cathepsin S inhibitor. Additional blood samples were then collected 3, 7, 12, 24, 48, and 72 h after administration of the cathepsin S inhibitor and PBMC enriched for Lip10 detection.

### Cathepsin S Activity in Healthy Human Volunteers Dosed with Cathepsin S Inhibitor

Healthy human volunteers were enrolled in a single-center, randomized, double-blind, placebo-controlled, single ascending dose study (www.ClinicalTrials.gov, identifier NCT02295332). The study was conducted in accordance with the Declaration of Helsinki, current International Conference on Harmonisation of Technical Requirements for Registration of Pharmaceuticals for Human Use (ICH) guidelines, and all applicable regulatory and ethical requirements. Written informed consent was obtained from all volunteers before the start of study procedures. The study protocol was approved by the Dutch ethics committee. The study employed an interleaved cohort design in which dosing was alternated between two cohorts, and each individual within a cohort received the study drug dosing on four occasions. Study participants received a single oral dose of RO5459072 (six volunteers) or placebo (two volunteers) per occasion. Assignment to either treatment group was randomized for each period of treatment. Blood samples were collected, in BD Vacutainer collection tubes containing sodium heparin, before drug administration and 2, 4, 6, 8, 12, 24, and 48 h after administration. PBMC were enriched from blood samples as described above, before being processed further for Lip10 detection. The study and clinical sample processing and analysis were carried out at PRA Health Sciences, Netherlands according to the methodology described here, after transfer of the method and successful completion of a validation procedure based on industry guidelines for bioanalytical method validation (data not shown).

### Detection of Intracellular Lip10 Accumulation

PBMC were pelleted and fixed with BD Phosflow Lyse/Fix Buffer (BD, Allschwill, Switzerland) for 10 min at 37°C. Immediately after fixation, the samples could be frozen in dry ice and stored at −80°C for up to 4 weeks without any impact on the stability of the markers detected, as determined by additional stability assessments (data not shown). Fixed cells were later permeabilized with BD Phosflow Perm Buffer IV (BD, Allschwill, Switzerland) for 20 min at room temperature, before being stained for 1 h at room temperature with unlabeled anti-Lip10 antibody in Cell Staining Buffer (BioLegend, London, UK). After initial assessment of the performance of the different clones available, 1 µg of 7B8 antibody per staining was chosen as the optimal condition for intracellular detection of Lip10 accumulation (data not shown). PE-conjugated, goat anti-rabbit IgG antibody (SouthernBiotech, Birmingham, AL, USA) in Cell Staining Buffer was then added to the cells and incubated for 30 min at room temperature. Finally, the cells were stained for 20 min at 4°C with a lineage antibody cocktail in Cell Staining Buffer containing PerCP-Cy5.5-conjugated anti-CD45 (BioLegend, London, UK), BV421-conjugated anti-CD3 (BioLegend, London, UK), APC-conjugated anti-CD14 (Beckman Coulter, Nyon, Switzerland), and Alexa Fluor 488-conjugated anti-CD20 (BD, Allschwill, Switzerland) antibodies. Stained samples were kept on ice until data acquisition.

### HLA-DR Surface Expression

PBMC were stained for 30 min at 4°C with an antibody mix in Cell Staining Buffer containing PerCP-conjugated anti-CD45 (BD, Allschwill, Switzerland), BV421-conjugated anti-CD14 (BD, Allschwill, Switzerland), APC-conjugated anti-CD19 (BD, Allschwill, Switzerland), and PE-conjugated anti-HLA-DR (R&D Systems, Abingdon, UK). Stained samples were kept on ice until data acquisition.

### Flow Cytometry and Data Analysis

Stained human samples were acquired on a BD FACSCanto II flow cytometer (BD, Allschwill, Switzerland). Compensation of all fluorochromes was performed using BD CompBeads (BD, Allschwill, Switzerland) according to the manufacturer’s instructions. The gating scheme used to analyze Lip10 data from PBMC samples within distinct lineage populations initially comprised a gate on CD45-positive events to select leukocytes, followed by doublet-excluding gates on forward scatter area v. height and side scatter area v. height, and a gate around all cells with normal size (forward scatter) and granular (side scatter) characteristics. Within this last gate, T cells were selected by drawing a gate around CD3-positive, CD20-negative events, and B cells with a gate around CD3-negative, CD20-positive events. Finally, monocytes were selected by drawing a gate around CD3-negative, CD20-negative events and, within this late gate, drawing a gate around CD14-positive events. In the case of cynomolgus monkeys, due to a lack of availability of suitable clones for staining CD45, CD3, and CD14 after fixation and permeabilization, the gating scheme was adapted to first exclude doublet events and gate on cells with normal size and granularity, as described above. In this last gate, T cells were then selected by drawing a gate around CD20-negative events with a low forward scatter, and B cells with a gate around CD20-positive events with a low forward scatter. In both types of sample, a sufficient number of cells were recorded to have a minimum of 500 B cells for each sample. Flow cytometry data analysis software FlowJo (FlowJo, Ashland, OR, USA) was used to further derive median fluorescence intensity (MFI) values for Lip10 (p10 MFI) of B cells and T cells in all samples, and of monocytes in human samples. Additionally, a Lip10 stain index for B cells was calculated for each sample by dividing the Lip10 MFI value of the B cell population by that of the T cell population. A Lip10 stain index for monocytes was also calculated for each human sample by dividing the Lip10 MFI value of the monocyte population by that of the T cell population. The gating scheme used to analyze HLA-DR data from PBMC samples comprised a gate on CD45-positive events to select leukocytes, followed by doublet-excluding gates on forward scatter area v. height and side scatter area v. height, and a gate around all cells with normal size (forward scatter) and granular (side scatter) characteristics. Within this last gate, monocytes were selected by drawing a gate around CD14-positive, CD19-negative events, and B cells with a gate around CD14-negative, CD19-positive events. MFI values for HLA-DR were derived for both monocytes and B cells in each sample. All statistical calculations, curve fitting, and derivation of EC50 values were performed using commercially available statistical software GraphPad Prism (GraphPad Software, La Jolla, CA, USA) or Spotfire (Tibco Software, Boston, MA, USA).

### Western Blotting

PBMC were enriched from human buffy coats obtained from the Swiss Red Cross Blood Donation Center (Basel, Switzerland) by gradient separation, as described for whole blood samples. An aliquot of PBMC was used for *ex vivo* treatment with cathepsin S inhibitor RO5459072 as described above. The remaining isolated PBMC were further processed to enrich B cells by mean of an EasySep Human B Cell Enrichment Kit (STEMCELL Technologies, Grenoble, France). Isolated B cells were then treated with either DMSO or 10µM cathepsin S inhibitor as described above. To serve as a positive control, cultured RAJI were incubated in parallel with isolated B cells under the same conditions. After incubation, cells were washed with PBS and pelleted before being lysed with 1X RIPA Buffer (Cell Signaling Technology Europe, Leiden, Netherlands) containing 1mM PMSF. Lysates were electrophoresed on a 4–12% polyacrylamide gel before protein transfer to a nitrocellulose membrane. The membrane was incubated with an anti-CD74 antibody (Abcam, Cambridge, UK) or Lip10 antibody followed by an HRP-conjugated goat anti-mouse antibody (SouthernBiotech, Birmingham, AL, USA). The membrane was developed with Lumi-Light Western Blotting Substrate (Roche Diagnostics, Mannheim, Germany). The membrane was further incubated with an anti-COX IV antibody (Abcam, Cambridge, UK) to serve as loading control, followed by an HRP-conjugated goat anti-mouse antibody and developed a second time as described above.

## Results

### Development of Lip10 Intracellular Accumulation Assay by Flow Cytometry

Monoclonal antibodies raised against Lip10 neoepitope antigens by immunization of rabbits were screened for their ability to detect intracellular Lip10 in a flow cytometry-based assay. To that purpose, RAJI cells were incubated in the presence of 0.1% DMSO (vehicle) or a cathepsin S inhibitor, known to lead to an intracellular accumulation of Lip10 as detected by western blotting (Figure 1B), and stained with individual clones after fixation and permeabilization. To minimize background signal under basal or cathepsin S inhibitor untreated conditions and to maximize the dynamic range of the assay, the objective was to identify a monoclonal antibody clone only recognizing Lip10 neoepitope, the novel c-terminal fragment created by proteolytic cleavage of protein Lip22, without binding to full length Ii or Lip22 precursor protein. Due to rapid turnover of Lip10 in the presence of active cathepsin S enzyme, a neoepitope-specific clone would therefore display low signal in the DMSO-treated controls and high signal in cathepsin S inhibitor-treated cells, resulting in a high stain index as calculated by dividing MFI of cathepsin S-treated/DMSO-treated cells. Of the 12 clones tested, 9 could reliably detect an accumulation of Lip10 in those cells, as seen by comparing the Lip10 MFI of cathepsin S inhibitor versus DMSO-treated RAJI cells (Figure 1C). Clone 7B8 proved to yield the best stain index increase upon Lip10 accumulation and was selected for further assay optimization, including the selection of fixation and permeabilization conditions and antibody titration (data not shown).

### Specific Detection of Lip10 Intracellular Accumulation in Professional APC Subsets

**Figure 2:**
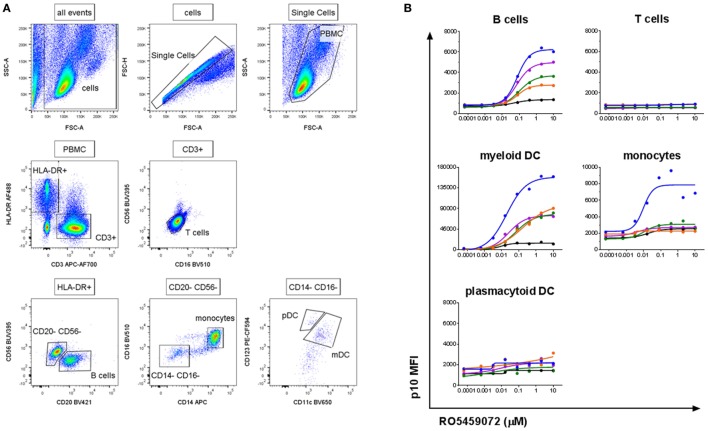
Detection of a dose-dependent Lip10 intracellular accumulation in professional antigen-presenting cell (APC) subsets. **(A)** Representative gating scheme used for Lip10 detection in immune subtypes with a flow cytometry-based assay. **(B)** Lip10 accumulation [p10 median fluorescence intensity (MFI)] in different immune subtypes was assessed by flow cytometry after *in vitro* incubation of PBMC with a serial titration of RO5459072. Data represent five individual healthy volunteers, identified by identical individual colored lines and dots across graphs. Curve fit was determined by non-linear regression using a variable slope model with GraphPad Prism software.

To verify the specificity of the antibody and assay for the detection of Lip10 accumulation in primary cells, PBMC from healthy human volunteers were incubated with serial dilutions of cathepsin S inhibitor. The gating applied to identify the specific PBMC subsets is depicted in Figure 2A. As expected, intracellular Lip10 accumulation could be detected in B cells within the PBMC population (Figure 2B). Blood-derived monocytes did only marginally upregulate Lip10 after incubation with inhibitor in four of five donors tested, although one of five donors exhibited strong Lip10 induction (Figure 2B). Surprisingly, blood dendritic cell subsets of myeloid (mDC) and plasmacytoid (pDC) origin displayed differential dependence on active cathepsin S for MHCII maturation. Whereas inhibition of cathepsin S in mDC resulted in strong upregulation of Lip10, pDC did not accumulate detectable Lip10 levels (Figure 2B). The method is specific as demonstrated by complete absence of Lip10 accumulation in T cells (Figure 2B). In conclusion, these results confirm published data claiming cathepsin S is the critical enzyme for MHCII maturation in B cells and mDC. These results also corroborate literature reports claiming redundancy through coexpression of CatL in non-IFNγ-activated monocytes/macrophages, although no donor variability has been reported previously (10). The absence of cathepsin S requirement for MHCII maturation in pDC is an interesting novel finding. Furthermore, the extent of Lip10 accumulation upon cathepsin S inhibition was found to be dose dependent.

### Robustness of B Cell Lip10 Assay As a Pharmacodynamic Readout

**Figure 3:**
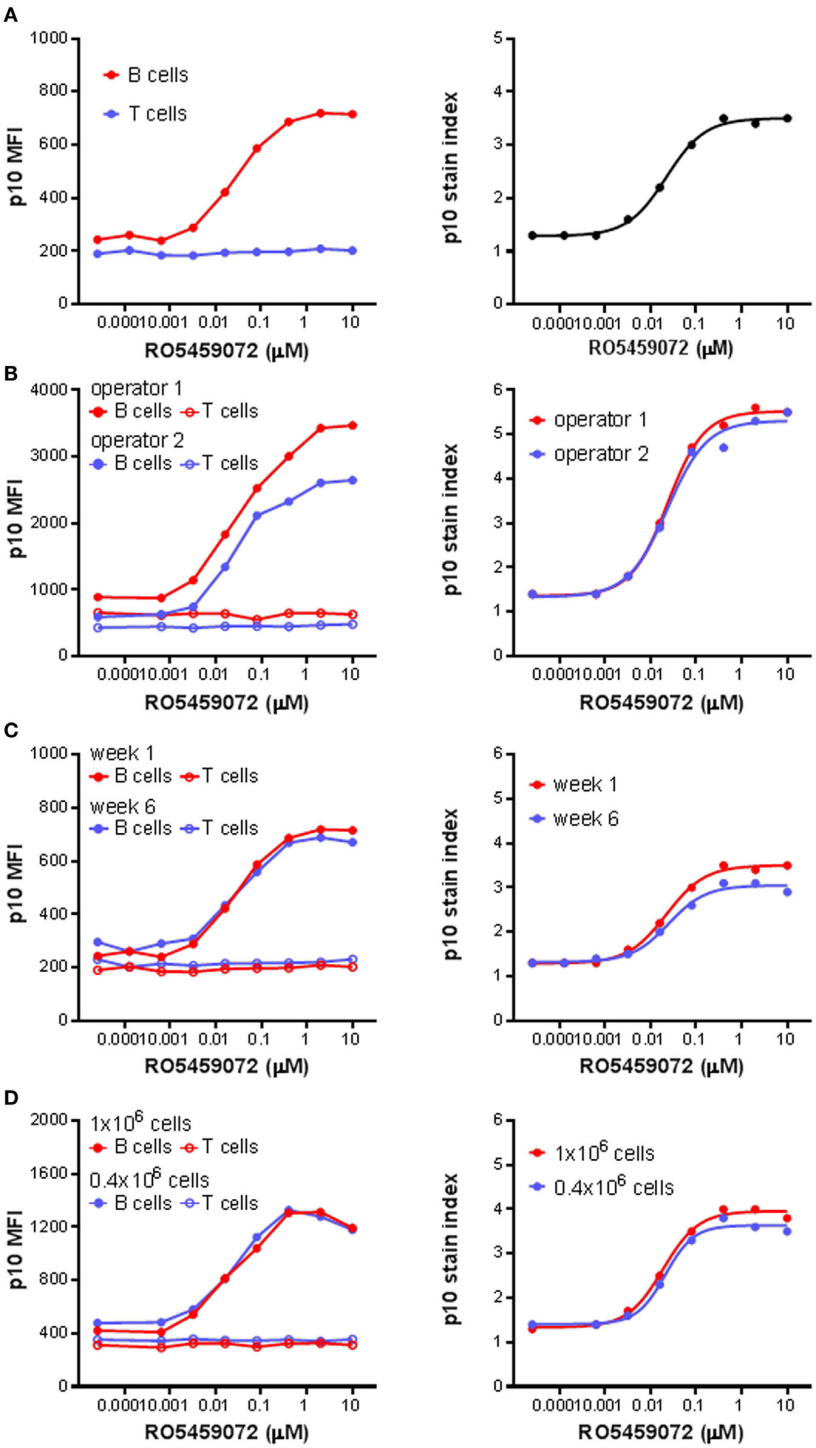
p10 stain index, a robust pharmacodynamic assay readout. **(A)** p10 stain index values were derived from p10 median fluorescence intensity (MFI) values of B cells of PBMC divided by p10 MFI values of T cells, which acted as an internal negative control. **(B)** p10 MFI values and derived p10 stain index values from identical samples of PBMC incubated with a serial titration of RO5459072 processed by two different operators. **(C)** Longitudinal comparison of p10 MFI values and derived p10 stain index values from samples of PBMC obtained from a single healthy volunteer 5 weeks apart and incubated with a serial titration of RO5459072. **(D)** Impact of variation in cell number incubated with a serial titration of RO5459072 on p10 MFI values and derived p10 stain index. Curve fits were determined by non-linear regression using a variable slope model with GraphPad Prism software.

Next, the Lip10 method was further developed for use as an *ex vivo* and *in vivo* pharmacodynamic monitoring readout focusing on Lip10 accumulation in B cells as surrogate marker for intracellular cathepsin S inhibition. For all subsequent experiments focusing on Lip10 in B cells, the gating scheme was modified compared to Figure 2A to make the identification of B cells independent of HLA-DR expression reduction potentially induced by treatment with cathepsin S inhibitor. Instead, using PBMCs as parental gate, a simple CD3^−^CD20^+^-gate was used to identify B cells and a CD3^+^CD20^−^ gate was used to identify T cells (not shown). Using the lack of cathepsin S expression and Lip10 accumulation in T cells as an internal negative control for normalization [Ref. (10) and Figure 2B], the Lip10 MFI values were transformed to a Lip10 stain index as a means to control for variability in staining between samples and facilitate data comparability (Figure 3A). When applied to a set of samples of PBMC incubated with a cathepsin S inhibitor and stained by different operators, the variability in fluorescence observed between operators in terms of Lip10 MFI values for B cells was not seen with the respective calculated Lip10 stain indexes, as illustrated by the overlap between the resulting fitted dose–response curves (Figure 3B). The established assay was found to show both repeatability and robustness. When the incubation of PBMC from a single healthy volunteer with a serial dilution of a cathepsin S inhibitor was repeated 5 weeks apart, the Lip10 accumulation measured under these same operating conditions was found to be very similar (CV <20%) on both occasions (Figure 3C). In addition, the Lip10 accumulation resulting from cathepsin S inhibition was not affected by the number of cells processed in the assay (Figure 3D) or the staining volumes (data not shown). These results demonstrated the method is sufficiently robust to qualify as a pharmacodynamic biomarker assay for application in a clinical study.

### Heterogeneity in Lip10 Accumulation upon Cathepsin S Inhibition of PBMC from Healthy Volunteers

**Figure 4:**
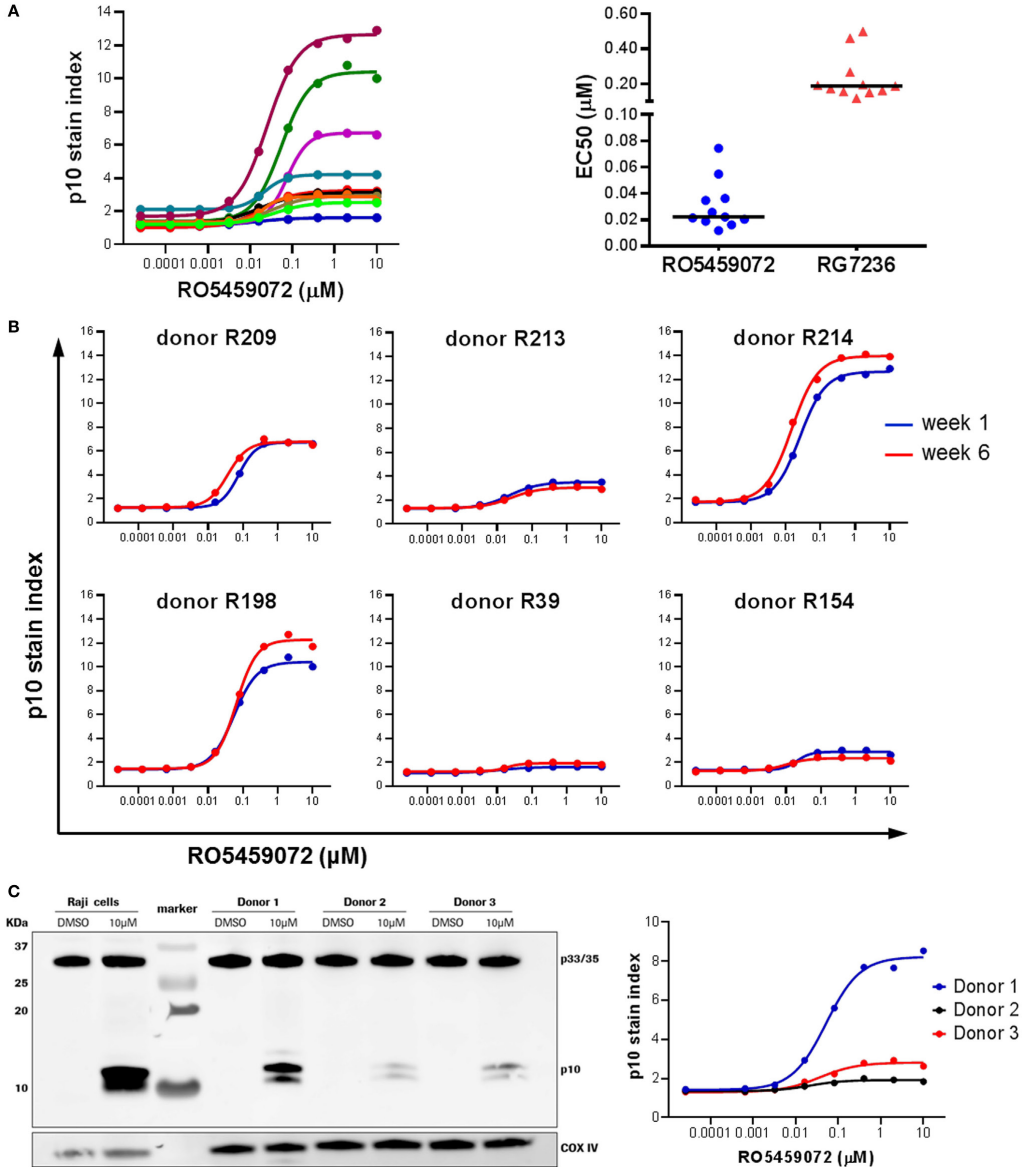
Heterogeneity in Lip10 accumulation upon cathepsin S-specific inhibition. **(A)** Lip10 accumulation in B cells of 11 healthy individuals after *in vitro* incubation of PBMC with a serial titration of cathepsin S inhibitor RO5459072. EC50 values were derived from curves fitted by non-linear regression using a variable slope model with GraphPad Prism software for two different cathepsin S inhibitors, RO5459072 and RG7236. **(B)** Longitudinal comparison of the heterogeneity in Lip10 accumulation in B cells of six healthy individuals. **(C)** Comparison of p10 stain index values obtained from three healthy individuals to p10 protein levels detected by western blotting. PBMC enriched from buffy coat were incubated *in vitro* with a serial titration of RO5459072 and Lip10 accumulation detected by flow cytometry with a Lip10 neoepitope antibody. In parallel, B cells enriched from the PBMC fraction were incubated *in vitro* with 10µM of RO5459072 or DMSO, and lysed for detection of Lip10 accumulation by western blotting with an anti-CD74 antibody. COX IV protein detection was used as a loading control.

Analysis of PBMC from a range of healthy volunteers incubated *ex vivo* in the presence of a serial dilution of cathepsin S inhibitor revealed interindividual heterogeneity in the maximal level of Lip10 accumulation in B cells upon inhibition of cathepsin S (Figure 4A). Volunteers could be broadly divided into two groups: low responders with Lip10 stain indexes up to 4 and high responders with Lip10 stain indexes greater than 4. The majority of the subjects fell into the low responder group. This heterogeneity was not specific to the cathepsin S inhibitor used, as comparison of two different inhibitors revealed the same heterogeneity in maximum Lip10 response (data not shown). While the maximum amount of Lip10 accumulated in B cells varied, the EC50 did not for the same compound, and the assay could reproducibly detect differences in terms of potency between the two compounds RO5459072 and RG7236 tested (Figure 4A). Importantly, baseline levels (untreated control conditions) were highly similar across individuals. Furthermore, repeated assessments showed that the assay yielded reproducible results, a clear indication of longitudinal stability (Figure 4B). To confirm that the results observed were not an artifact of the technology employed, the assay was carried out in parallel with western blotting, a more commonly used method to detect Lip10 accumulation in cells albeit requiring B cell enrichment prior to sample processing. As seen in the comparison of three healthy volunteers with differing levels of Lip10 accumulation upon cathepsin S inhibition, both western blotting and flow cytometry-based assay described here produced similar observations (Figure 4C). When ranked by band intensity or Lip10 stain index, the donor 1 was found to be a high responder, while donors 2 and 3 were low responders, with B cells from donor 3 accumulating marginally more Lip10 than those from donor 2. In order to test the specificity of the Lip10-recognizing antibody, this reagent was directly tested in a western blot (Figure S1 in Supplementary Material). The antibody indeed recognized a region above 10 kDa corresponding to Lip10 in stimulated cells only and did not detect full length invariant chain or any other protein, thereby demonstrating specificity.

### Lip10 As a Pharmacodynamic Biomarker in Cynomolgus Monkeys Dosed with RO5459072

**Figure 5:**
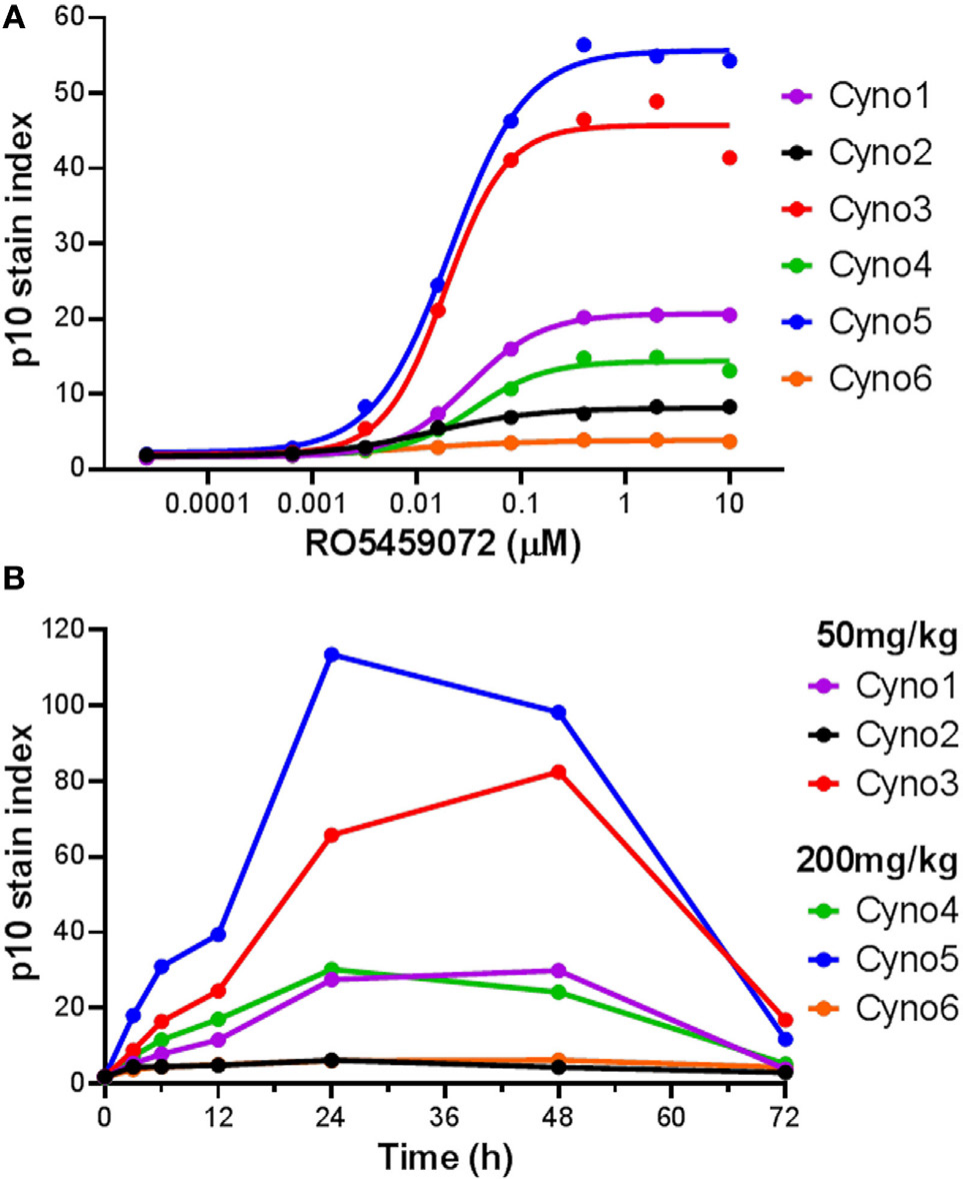
Detection of Lip10 accumulation by flow cytometry in cynomolgus monkeys after oral administration of RO5459072. **(A)** Lip10 accumulation in B cells of six cynomolgus monkeys at baseline, after *in vitro* incubation of PBMC with a serial titration of cathepsin S inhibitor RO5459072. Curve fit was determined by non-linear regression using a variable slope model with GraphPad Prism software. **(B)** Detection of Lip10 accumulation in circulating B cells from six cynomolgus monkeys, measured by flow cytometry in PBMC enriched from whole blood samples taken at baseline (0 h) and after oral administration of 50 or 200 mg/kg cathepsin S inhibitor RO5459072 (3–72 h).

The utility of a Lip10 flow cytometry-based assay as a readout of cathepsin S inhibitor pharmacodynamic activity in treated animals *in vivo* was tested by comparing the responses in cynomolgus monkeys administered either a 50 or 200 mg/kg single oral dose of cathepsin S inhibitor RO5459072. Prior to compound administration, a blood sample was taken from each animal for PBMC enrichment and *ex vivo* incubation with a serial dilution of the cathepsin S inhibitor. Detection of Lip10 accumulation in B cells revealed the same degree of heterogeneity in terms of interindividual maximal accumulation as that seen for healthy human volunteers, albeit with a different degree of Lip10 stain index magnitude (Figure 5A). Derived EC50 values for the compound (21.5 ± 9.4nM, *n* = 6) were found to be in range with that of blood samples obtained from human subjects tested with the same compound (31.5 ± 18.6nM, *n* = 10). Dosing of monkeys with RO5459072 resulted in an intracellular Lip10 accumulation in B cells in the blood of all six animals *in vivo*, as measured by the flow cytometry assay (Figure 5B). Lip10 was found to peak at around 24 h post administration, and return to basal levels at 72 h post administration. No evidence of a relationship between the dose administered and the level of response in terms of Lip10 accumulation could be found, but a retrospective analysis revealed actually both dosing groups reached saturating levels of target inhibition and therefore no dose-dependent PD effect would be expected in this experiment. However, within each treatment group the ranking of animals based on the Lip10 stain index (Figure 5B) corresponded to that observed in the *ex vivo* assessment (Figure 5A). Taken together, these results establish that all monkeys dosed with RO5459072 were responsive to the inhibitor, confirming that cathepsin S inhibitor reached its intended target after oral administration. Furthermore, *ex vivo* incubation of cynomolgus monkey PBMC with the compound could predict relative interindividual magnitude of *in vivo* pharmacodynamic effects. An empirical indirect pharmacokinetic and pharmacodynamic model was established to relate the EC50/Emax obtained from fresh monkey blood incubated with cathepsin S inhibitor to the IC50/Imax of cathepsin S measured *in vivo* after oral administration (not shown). The model showed relative good confidence of an existing relationship, predictive of cathepsin S inhibition *in vivo*, despite the limited amount of dataset gained from the *in vivo* experiment. These data confirmed the potential applicability of Lip10 as pharmacodynamic biomarker for dose selection in humans.

### Immunopharmacologic Monitoring of RO5459072 Pharmacodynamics in a Single Dose Study

**Figure 6:**
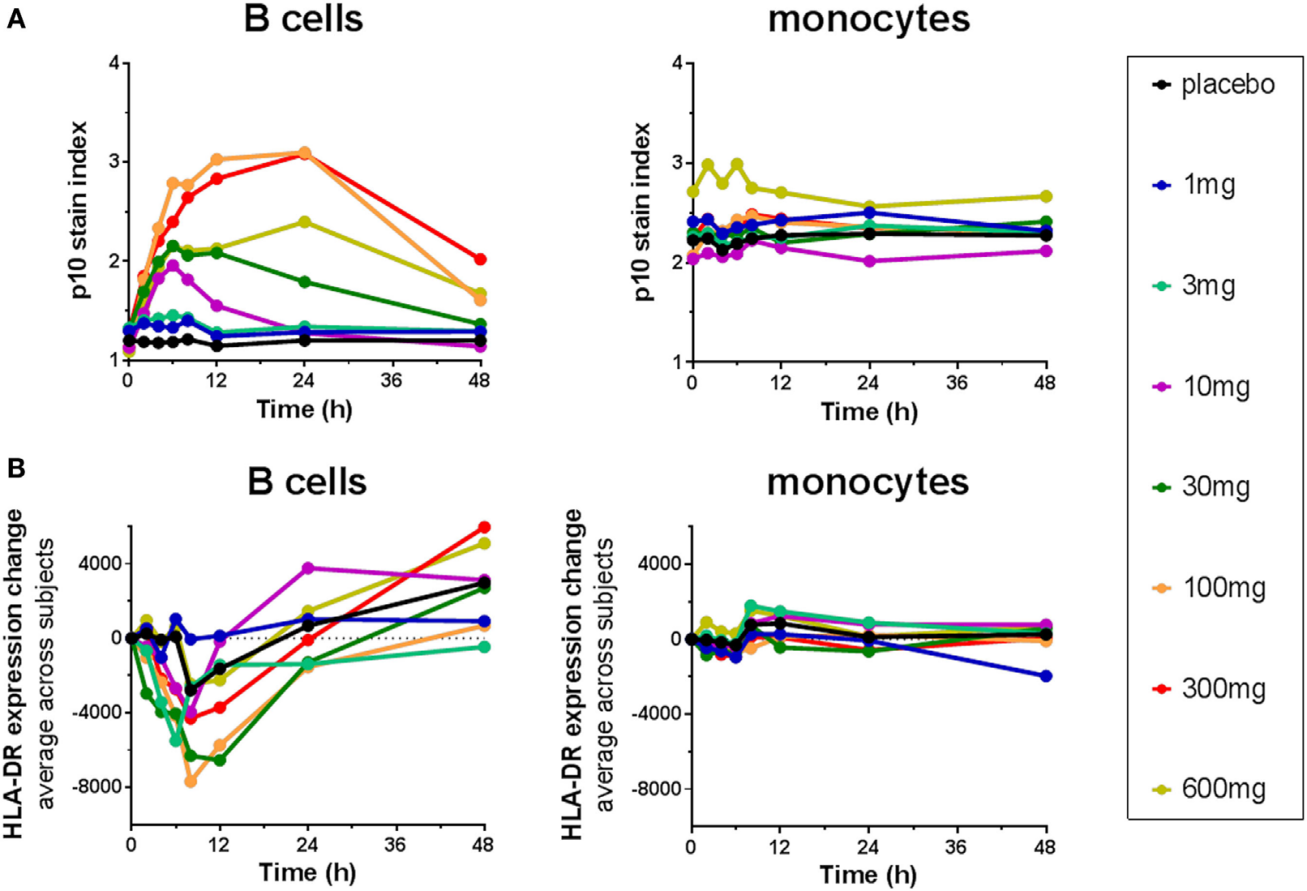
Cathepsin S inhibitor RO5459072 induced Lip10 accumulation and major histocompatibility complex class II expression reduction in B cells but not monocytes of healthy volunteers. Healthy volunteers enrolled in a single ascending dose clinical study of RO5459072 were given a single oral dose of the cathepsin S inhibitor ranging from 1 to 600 mg or a placebo control. Intracellular p10 stain index **(A)** and HLA-DR surface expression **(B)** were measured in circulating B cells and monocytes from PBMC enriched from whole blood samples taken at baseline (0 h) and after administration of the inhibitor or placebo (2–48 h). Data represent averages of six subjects per treatment group.

**Figure 7:**
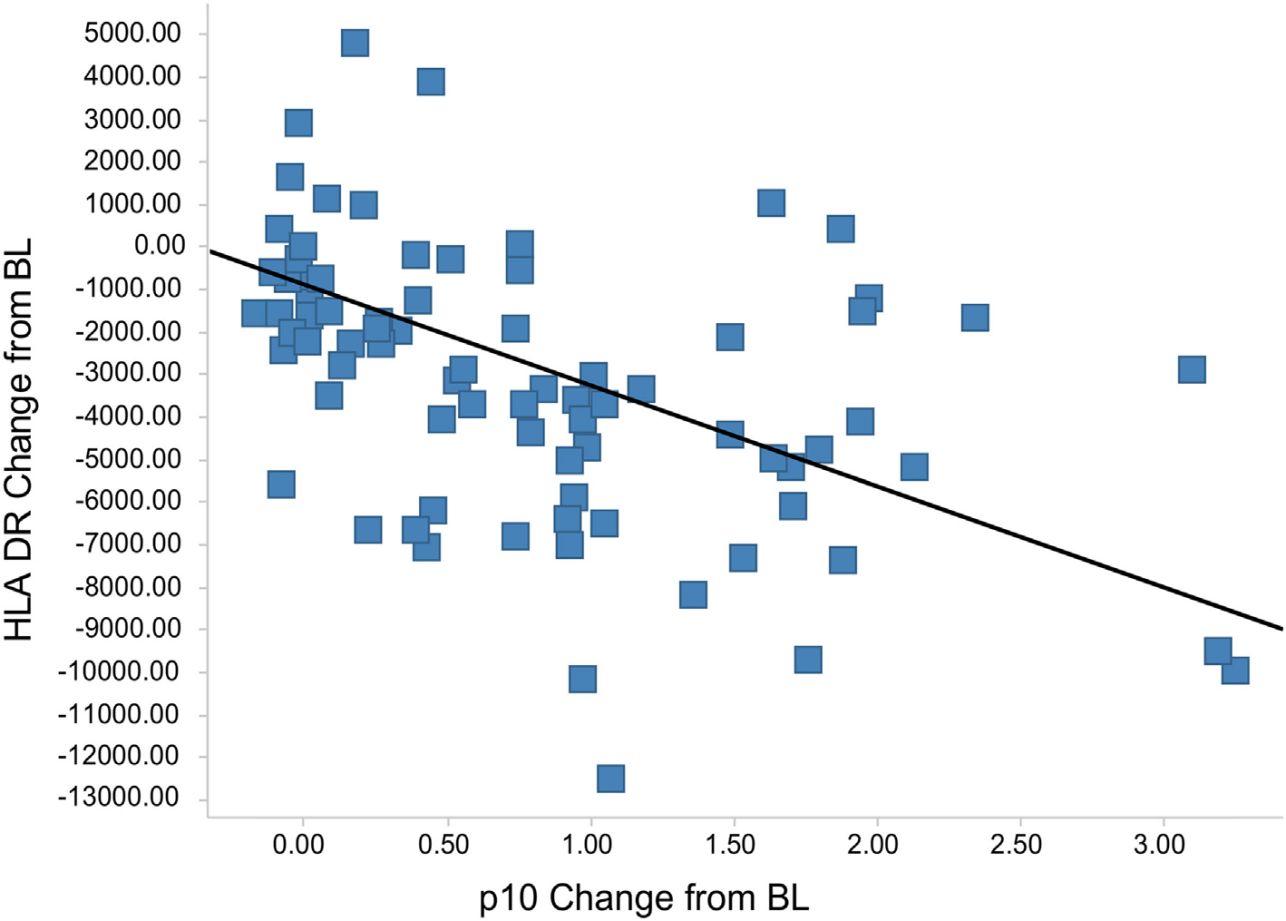
Lip10 accumulation correlates with decrease in major histocompatibility complex class II (MHCII) surface expression in clinical study participants. A linear regression analysis was performed with Lip10 accumulation and MHCII expression, both calculated as change from baseline, before dosing at time-point = 0 h. The analysis was restricted to time-points before dosing and 8 and 12 h after dosing to restrict the analysis to time-points with large enough overlap in the kinetics of both markers (*r*^2^ = 0.35, *p* < 0.001 × 10^−9^).

The Lip10 flow cytometry-based assay was included as secondary endpoint assessment in a first-in-human clinical study whose primary objective was to investigate the safety and tolerability of cathepsin S inhibitor RO5459072 in healthy human volunteers. Lip10 was intended to provide a measurement of target engagement to help characterize the dose–response relationship as well as to enable identification of the optimal biological dose required for maximum target inhibition. Human volunteers were given a single oral dose of either placebo (two subjects per cohort) or RO5459072 (six subjects per cohort), ranging from 1 to 600 mg, in increasing amounts over a total of eight dosing periods of treatment. All doses tested were tolerated similarly to placebo and showed linear pharmacokinetics across the majority of the dose range. In all individuals dosed with RO5459072, a response in the form of Lip10 accumulation could be detected in B cells (Figure 6A and individual plots in Figure S2 in Supplementary Material). A clear increase in intracellular Lip10 was observed with doses of 10 mg and above, which peaked at 6–24 h after the cathepsin S inhibitor was administered before returning to near basal levels at 48-h post administration. Lip10 accumulation appeared to be dose-dependent, as indicated by Lip10 stain index values, and the minimum dose leading to the maximal observed effect was 100 mg. Average Lip10 stain index peak levels at doses of 100, 300, and 600 mg were similar, although a trend for lower maximum increase in LIP10 was observed at the highest dose tested (Lip10 stain index of 3.10 ± 0.84 SD, 3.08 ± 0.41 SD, and 2.40 ± 0.78 SD, respectively). Effect of drug on peak Lip10 stain index comparing the results from subjects receiving placebo versus subjects receiving 100 mg was highly significant (*p* = 0.0005, Kruskal–Wallis). There was no statistical difference in the effect on Lip10 stain index comparing 100 versus 300 mg dose (*p* = 0.15, Kruskal–Wallis) or 100 versus 600 mg dose (*p* = 0.20, Kruskal–Wallis). In comparison to B cells, no such observations could be made in monocytes (Figure 6A). In those cells, Lip10 stain index values remained close to baseline throughout the duration of the time-course after treatment and for all doses tested. These results show that the Lip10 flow cytometry-based assay provided confirmation of the target engagement in B cells by the cathepsin S inhibitor RO5459072 in healthy subjects receiving therapeutic doses *in vivo*. The pharmacodynamic measurements also provided information on dose exposure and resulting effects. In line with published data reporting inhibition of cathepsin S lead to diminished antigen presentation in professional APCs, a reduction of up to 44% in expression of MHCII on the cell surface of B cells analyzed from study participants was detected, although the regulation of MHCII *in vivo* was not dose dependent (Figure 6B). MHCII surface expression determined by flow cytometry reached trough levels 8 h after dosing. The maximum decrease compared to placebo was measured in the 100 mg dose group with a mean fluorescence intensity averaging 16,834 ± 4,109 SD and 9,931 ± 3,002 SD, respectively. This difference was statistically significant (*p* = 0.0017). Surprisingly, MHCII surface expression was not significantly decreased compared to placebo in the 300 and 600 mg dosing groups (MHCII MFI 15,443 ± 4,064 SD and 13,686 ± 4,077 SD, *p* = 0.8 and *p* = 0.16 Kruskal–Wallis). The reduced expression of MHCII observed *in vivo* was consistent with *in vitro* data showing a dose-dependent decrease between 10 and 40% of MHCII signal compared to untreated controls on human B cells treated with RG7236, another cathepsin S inhibitor not further pursued for clinical development (Figure S3 in Supplementary Material). As expected based on the low to absent Lip10 accumulation in monocytes *in vitro* described earlier, Lip10 also remained at baseline levels on monocytes *in vivo* (Figure 6A). Furthermore, lack of treatment-induced increase of Lip10 within monocytes was concordant with stable monocyte MHCII expression levels *in vivo* (Figure 6B). To investigate whether the degree of B cell intracellular Lip10 accumulation correlated with reduced B cell MHCII cell surface expression levels, a linear regression analysis was performed using clinical data from all study participants receiving cathepsin S inhibitor (Figure 7) at baseline and at Lip10 peak levels (8 and 12 h after dosing). Although a clear trend is shown that high Lip10 accumulation correlated with a strong decrease in MHCII surface expression levels, the low *r*^2^ value of 0.35 indicates that Lip10 accumulation alone is a relatively poor predictor of MHCII expression decrease. In conclusion, there must be additional factors contributing to decreased MHCII surface expression under cathepsin S inhibitory conditions *in vivo*. Possible explanations that require further investigation are measurement imprecision in a clinical setting (technical variability), genotype, particularly HLA haplotype of study subjects and different kinetics of MHCII surface expression and Lip10 intracellular accumulation. Overall, these data confirm the specificity of Lip10 induction as a surrogate marker for inhibition of cathepsin S *in vivo* in humans. This accumulation coincides with a reduction of MHCII surface expression on susceptible antigen-expressing cells in treated human subjects *in vivo*, and a trend exists for stronger MHCII surface expression reduction in individuals in which high Lip10 accumulation can be detected.

## Discussion

Here, we show that a novel neoepitope-specific flow cytometry-based assay was able to measure the cell-specific accumulation of Lip10 as a surrogate for impaired MHCII maturation and antigen presentation. Inhibition of cathepsin S by a specific inhibitor, RO5459072, in PBMC *ex vivo* resulted in accumulation of Lip10 in B cells and mDC, but not in pDC and only to a minor degree in monocytes. Application of the method to PBMC from cynomolgus monkeys treated with RO5459072 confirmed the accumulation of Lip10 *in vivo* and the interindividual heterogeneity of the pharmacodynamic response observed *in vitro*. Finally, dosing of RO5459072 in healthy human volunteers showed a dose-dependent increase in Lip10 index, confirming target engagement and demonstrating desired pharmacologic inhibition *in vivo*. These results will help guide subsequent clinical studies investigating the pharmacokinetic and pharmacodynamic relationship of cathepsin S inhibitor after multiple dosing.

The role of antigen-specific activation of CD4^+^ helper T cells and downstream activation of effector mechanisms such as autoantibody production by B cells in the pathogenesis of a number of autoimmune diseases such as RA and Sjögren’s syndrome is well established. This has led to an interest in interfering with the antigen-specific activation of T cells as a means to suppress the autoimmune response. The central role of cathepsins in the MHC-mediated peptide presentation machinery of professional APC makes them a preferential target for the development of inhibitory compounds to limit autoantigen presentation. An appealing aspect of inhibiting cathepsin S in MHCII presentation could be the specificity of this treatment approach. Cathepsin S is not required for loading MHCI molecules; therefore, inhibition does not affect CD8^+^ T cell activation, sparing the cellular adaptive immunity arm. Unlike commonly used broad spectrum immunosuppressants such as steroids, inhibition of cathepsin S could therefore be a more selective immunoregulatory agent with fewer side effects. Several studies have confirmed the viability of inhibiting cathepsin S in preclinical models (14–16, 21).

An interesting finding of testing Lip10 accumulation in healthy volunteer-derived PBMCs after *ex vivo* incubation with cathepsin S inhibitors is the significant heterogeneity in maximum levels of Lip10 detectable in B cells and mDC, while the half-maximum efficacious concentration (EC50) remained stable. Heterogeneity in Lip10 levels in B cells upon cathepsin S inhibition *ex vivo* was also found in cynomolgus monkeys. Again, the EC50 remained consistent among animals and between cynomolgus monkey and human subjects. To our knowledge, the variability in Lip10 maximum accumulation has never previously been described for cathepsin S-specific inhibitors. This heterogeneity was found to be reproducible longitudinally using inhibitors of different potency, and it could be confirmed by western blotting. Western blotting, however, showed no difference in the precursor Ii levels present in B cells among subjects. An explanation for this interindividual heterogeneity could be the HLA status influencing maximum accumulation levels of Lip10 due to different binding affinities between HLA molecule and p10. Although this possibility cannot be completely ruled out, it seems unlikely that Lip10 degradation is more efficient in an HLA-free compared to an HLA-bound state under fully saturated cathepsin S target-inhibiting conditions. As donor heterogeneity was observed even at full cathepsin S inhibitory concentrations, this explanation seems unlikely. In line with this, an exploratory analysis of the clinical data did not indicate a marked effect of HLA haplotype on the response to RO5459072 (data not shown); however, the size of the dataset was limited, so that conclusions cannot be made based on these results. Another possible explanation would be inter-donor differences in the expression or activity of other enzymes involved in the Ii proteolytic cascade, which could lead to a partial compensation for the lack of cathepsin S activity. A combination of an individual’s immune status, environmental, and genetic factors may influence the set of cathepsins coexpressed in an individual. See also discussion below about IFNγ-specific effects influencing macrophage dependency on cathepsin S for MHCII maturation. Taken together, partial compensation by low-level expression of other enzymes such as cathepsin L in certain donors seems a likely explanation for the observed variability on maximum Lip10 accumulation that requires further investigation. Of note, the maximal Lip10 stain index observed in the *ex vivo* stimulation correlated well with pharmacodynamic effects observed after dosing *in vivo* both in cynomolgus monkey and human subjects. A resulting possible application of the Lip10 method could therefore be the employment as a predictive biomarker, postulating individuals with high levels of *in vitro* Lip10 accumulation could respond more favorably to cathepsin S inhibition, an interesting hypothesis requiring further clinical validation.

The data presented here confirm the non-redundant role of cathepsin S for MHCII maturation in mDC and B cells, both professional APC types known to play major roles in autoimmunity. In contrast, pDC were not affected by cathepsin S inhibition. Some degree of interindividual heterogeneity seems to exist regarding blood-derived monocytes, with approximately one in five donors exhibiting significant Lip10 accumulation also in monocytes, although this observation was restricted to *in vitro* testing conditions. Interestingly, IFNγ-activated monocytes/macrophages undergo a switch in cathepsin activity, which renders their MHCII maturation process again susceptible to cathepsin S inhibition [internal data, not shown, and Ref. (10, 22, 23)]. Therefore, the inhibition of cathepsin S may be particularly effective in treating inflammatory Th1-driven, mDC or B cell-driven autoimmune processes, such as psoriasis or multiple sclerosis, while at the same time preserving tolerogenic pDC function (24).

In addition to its valuable application as a pharmacodynamic biomarker assay and for cathepsin S inhibitor potency characterization, the method described here is a powerful research tool to further investigate the role of cathepsin S in MHCII maturation in other cell types not characterized so far. The potential to perform single cell analysis and multiplexing by flow cytometry allows the analysis of professional APC subtypes with low frequencies, a prohibitive factor for the application of conventional Lip10 western blot analysis. This includes studying the influence of differentiation stage and tissue location on cathepsin S-dependent MHCII maturation in DC subsets, such as Langerhans cells, or the influence of Th2 stimuli in macrophages.

A high attrition rate is one of the major challenges in pharmaceutical development. One of the key factors contributing to a high failure rate in phase II proof-of-concept studies is the lack of demonstrated target engagement and pharmacodynamics effect (17, 25). The successful clinical development of small molecule inhibitors requires a robust pharmacodynamic biomarker assay amenable to the scale and regulatory requirements of clinical studies. The pharmacodynamic assay described here enabled internal decision-making by demonstrating appropriate target inhibition, establishing pharmacokinetic and pharmacodynamic correlation and by using Lip10 biomarker data as a surrogate endpoint for clinical benefit. In line with this concept, the demonstration of cathepsin S inhibition *in vivo* within acceptable safety margins represented an early successful proof of mechanism and therefore increased confidence in the pharmacological potency and development potential of the molecule and target.

In conclusion, we have developed a new pharmacodynamic biomarker assay specific for Lip10 neoepitope to support the development of therapeutic compounds targeting invariant chain proteolysis in professional APCs. This assay enabled the characterization of a cathepsin S inhibitor in preclinical and clinical studies, confirming target engagement *in vivo*. The resulting ability to reproducibly quantify the pharmacokinetic and pharmacodynamic relationship in human studies is the foundation for future clinical pharmacology and efficacy studies of RO5459072.

## Ethics Statement

Human blood samples from healthy volunteers were collected under the Blood Donation for Research Purposes program at F. Hoffmann-La Roche, Basel, Switzerland. Written informed consent was obtained from all donors. Experiments were conducted in accordance with the Declaration of Helsinki and all applicable regulatory and ethical requirements. Cynomolgus blood samples were drawn from adult *Macaca fascicularis* monkeys (Bioprim, Baziege, France), housed and cared for according to the Swiss Animal Welfare Act and Ordinance. The work described here was carried out in accordance with the EU directive 2010/63/EU for animal experiments. Healthy human volunteers were enrolled in a single-center, randomized, double-blind, placebo-controlled, single ascending dose study (www.ClinicalTrials.gov, identifier NCT02295332). The study was conducted in accordance with the Declaration of Helsinki, current International Conference on Harmonisation of Technical Requirements for Registration of Pharmaceuticals for Human Use (ICH) guidelines, and all applicable regulatory and ethical requirements. Written informed consent was obtained from all volunteers before the start of study procedures. The study protocol was approved by the Dutch ethics committee.

## Author Contributions

The authors DB, SN, BR, and MM conceived the study and experiments. MT and BE developed reagents, methods, as well as performed experiments and data analysis with help from BR, AS, and PT. TS and CP conceived the cynomolgus study and experiments. DB and SN conceived the clinical single ascending dose study design in healthy volunteers. MG conceived and supervised the monoclonal antibody reagent development to obtain Lip10-specific reagents. MT and BR wrote the manuscript with input from all authors.

## Conflict of Interest Statement

The authors, MT, DB, SN, MM, TS, AS, BE, PT, CP, and BR, are employed by Hoffman-La Roche Ltd. and may own stocks issued by F. Hoffman-La Roche Ltd.

## References

[1] Neurath MF Finotto S Glimcher LH. The role of Th1/Th2 polarization in mucosal immunity. Nat Med (2002) 8:567–73. 10.1038/nm0602-567 12042806

[2] Sudzius G Mieliauskaite D Butrimiene I Siaurys A Mackiewicz Z Dumalakiene I Activity of T-helper cells in patients with primary Sjögren’s syndrome. In Vivo (2013) 30:263–8. 23422488

[3] Noack M Miossec P. Th17 and regulatory T cell balance in autoimmune and inflammatory diseases. Autoimmun Rev (2014) 13:668–77. 10.1016/j.autrev.2013.12.004 24418308

[4] Chastain EML Duncan DAS Rodgers JM Miller SD. The role of antigen presenting cells in multiple sclerosis. Biochim Biophys Acta (2011) 1812:265–74. 10.1016/j.bbadis.2010.07.008 20637861 PMC2970677

[5] Engel P Gómez-Puerta JA Ramos-Casals M Lozano F Bosch X. Therapeutic targeting of B cells for rheumatic autoimmune diseases. Pharmacol Rev (2011) 63:127–56. 10.1124/pr.109.002006 21245206

[6] Kulkarni OP Anders H-J. Lupus nephritis. How latest insights into its pathogenesis promote novel therapies. Curr Opin Rheumatol (2012) 24:457–65. 10.1097/BOR.0b013e328354c877 22810362

[7] Guagliardi LE Koppelman B Blum JS Marks MS Cresswell P Brodsky FM. Co-localization of molecules involved in antigen processing and presentation in an early endocytic compartment. Nature (1990) 343(6254):133–9. 10.1038/343133a0 2404209

[8] Blum JS Wearsch PA Cresswell P. Pathways of antigen processing. Annu Rev Immunol (2013) 31:443–73. 10.1146/annurev-immunol-032712-095910 23298205 PMC4026165

[9] Honey K Rudensky AY. Lysosomal cysteine proteases regulate antigen presentation. Nat Rev Immunol (2003) 3:472–82. 10.1038/nri1110 12776207

[10] Hsing LC Rudensky AY. The lysosomal cysteine proteases in MHC class II antigen presentation. Immunol Rev (2005) 207:229–41. 10.1111/j.0105-2896.2005.00310.x 16181340

[11] Riese RJ Wolf PR Brömme D Natkin LR Villadangos JA Ploegh HL Essential role for cathepsin S in MHC class II-associated invariant chain processing and peptide loading. Immunity (1996) 4:357–66. 10.1016/S1074-7613(00)80249-6 8612130

[12] Nakagawa TY Brissette WH Lira PD Griffiths RJ Petrushova N Stock J Impaired invariant chain degradation and antigen presentation and diminished collagen-induced arthritis in cathepsin S null mice. Immunity (1999) 10:207–17. 10.1016/S1074-7613(00)80021-7 10072073

[13] Shi GP Villadangos JA Dranoff G Small C Gu L Haley KJ Cathepsin S required for normal MHC class II peptide loading and germinal center development. Immunity (1999) 10(2):197–206. 10.1016/S1074-7613(00)80020-5 10072072

[14] Thurmond RL Sun S Sehon CA Baker SM Cai H Gu Y Identification of a potent and selective noncovalent cathepsin S inhibitor. J Pharmacol Exp Ther (2004) 308:268–76. 10.1124/jpet.103.056879 14566006

[15] Baugh M Black D Westwood P Kinghorn E McGregor K Bruin J Therapeutic dosing of an orally active, selective cathepsin S inhibitor suppresses disease in models of autoimmunity. J Autoimmun (2011) 36:201–9. 10.1016/j.jaut.2011.01.003 21439785

[16] Rupanagudi KV Kulkarni OP Lichtnekert J Darisipudi MN Mulay SR Schott B Cathepsin S inhibition suppresses systemic lupus erythematosus and lupus nephritis because cathepsin S is essential for MHC class II-mediated CD4 T cell and B cell priming. Ann Rheum Dis (2015) 74(2):452–63. 10.1136/annrheumdis-2013-203717 24300027

[17] Workman P. How much gets there and what does it do? The need for better pharmacokinetic and pharmacodynamic endpoints in contemporary drug discovery and development. Curr Pharm Des (2003) 9:891–902. 10.2174/1381612033455279 12678873

[18] Spinella DG Biomarkers in clinical drug development: realizing the promise. Biomark Med (2009) 3(6):667–9. 10.2217/bmm.09.61 20477703

[19] Hilpert H Mauser H Humm R Anselm L Kuehne H Hartmann G Identification of potent and selective cathepsin S inhibitors containing different central cyclic scaffolds. J Med Chem (2013) 56(23):9789–801. 10.1021/jm401528k 24224654

[20] Seeber S Ros F Thorey I Tiefenthaler G Kaluza K Lifke V A robust high throughput platform to generate functional recombinant monoclonal antibodies using rabbit B cells from peripheral blood. PLoS One (2014) 9(2):e86184. 10.1371/journal.pone.0086184 24503933 PMC3913575

[21] Figueiredo JL Aikawa M Zheng C Aaron J Lax L Libby P Selective cathepsin S inhibition attenuates atherosclerosis in apolipoprotein E-deficient mice with chronic renal disease. Am J Pathol (2015) 185(4):1156–66. 10.1016/j.ajpath.2014.11.026 25680278 PMC4380840

[22] Nakagawa T Roth W Wong P Nelson A Farr A Deussing J Cathepsin L: critical role in II degradation and CD4 T cell selection in the thymus. Science (1998) 280(5362):450–3. 10.1126/science.280.5362.450 9545226

[23] Beers C Honey K Fink S Forbush K Rudensky A. Differential regulation of cathepsin S and cathepsin L in interferon gamma-treated macrophages. J Exp Med (2003) 197(2):169–79. 10.1084/jem.20020978 12538657 PMC2193812

[24] Amodio G Gregori S. Dendritic cells a double-edge sword in autoimmune responses. Front Immunol (2012) 3:233. 10.3389/fimmu.2012.00233 22876246 PMC3410601

[25] Morgan P Van Der Graaf PH Arrowsmith J Feltner DE Drummond KS Wegner CD Can the flow of medicines be improved? Fundamental pharmacokinetic and pharmacological principles toward improving phase II survival. Drug Discov Today (2012) 17(9–10):419–24. 10.1016/j.drudis.2011.12.020 22227532

