# Supplementary material for: Pharmacodynamic Monitoring of RO5459072, a Small Molecule Inhibitor of Cathepsin S
Source: Front Immunol. 2017 Jul 17;8:806. doi: 10.3389/fimmu.2017.00806 (PMC5512459; doi:10.3389/fimmu.2017.00806)
Supplement: Supplementary file 1 [file Data_Sheet_1.DOCX]

Supplementary Material

Pharmacodynamic Monitoring of RO5459072, a Small Molecule Inhibitor of Cathepsin S

Michel Theron^1^, Darren Bentley^2^, Sandra Nagel^2^, Marianne Manchester^1^, Thomas Schindler^3^, Ana Silva^1^, Barbara Ecabert^1^, Priscila Teixeira^1^, Camille Perret^1^, Bernhard Reis^1*^

^1^ Roche Pharma Research and Early Development, Pharmaceutical Sciences, Roche Innovation Center Basel, F. Hoffmann-La Roche Ltd, Grenzacherstrasse 124, 4070 Basel, Switzerland

^2^ Roche Pharma Research and Early Development, Clinical Pharmacology, Roche Innovation Center Basel, F. Hoffmann-La Roche Ltd, Grenzacherstrasse 124, 4070 Basel, Switzerland

^3^ Roche Pharma Research and Early Development, Immunology, Inflammation and Infectious Diseases, Roche Innovation Center Basel, F. Hoffmann-La Roche Ltd, Grenzacherstrasse 124, 4070 Basel, Switzerland

*Corresponding author

Correspondence

Bernhard Reisbernhard.reis@roche.com

# Supplementary Figures and Tables

## Supplementary Figures


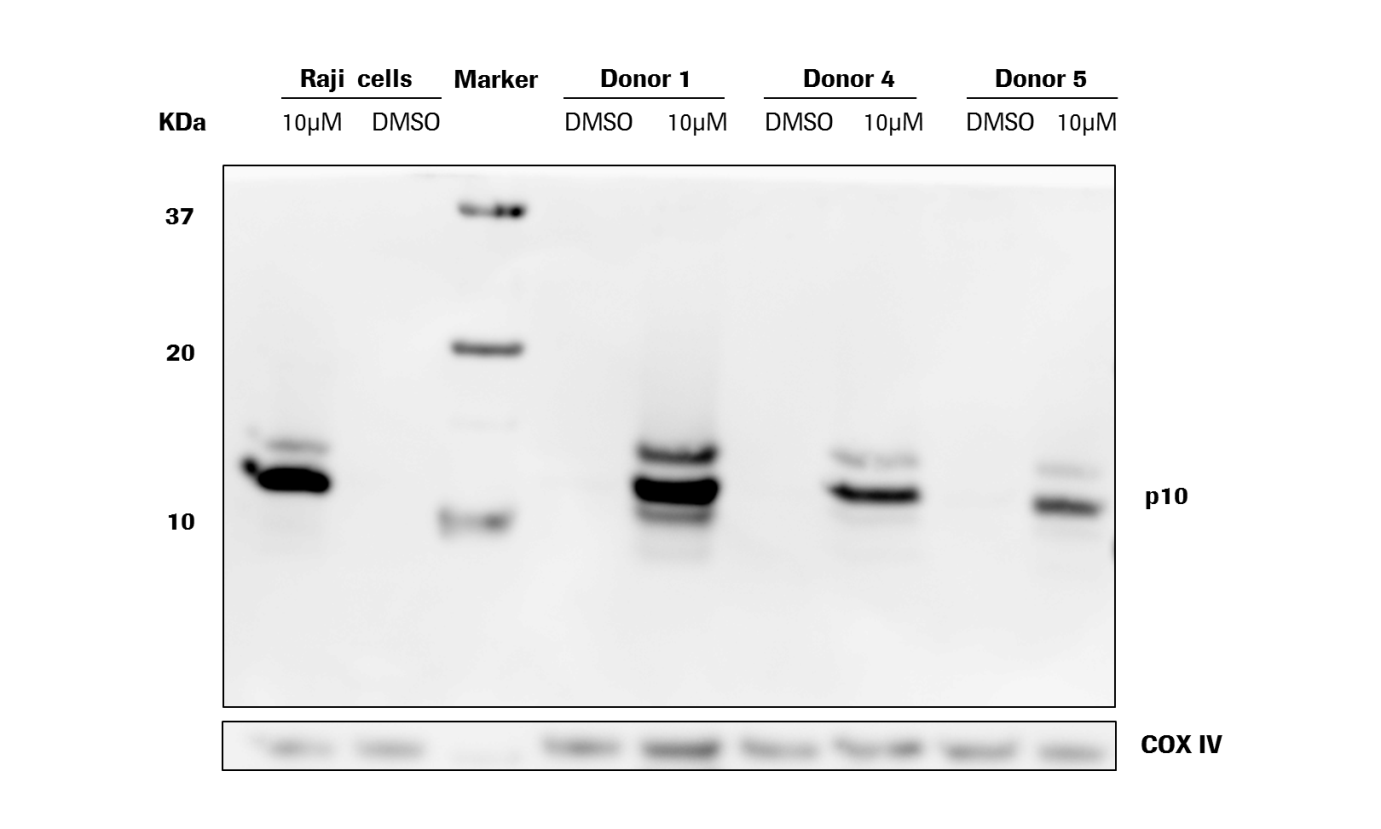


**Supplementary Figure 1** Specificity of Lip10 recognizing antibody demonstrated by Western blot. Raji cells or B cells enriched from buffy coats were incubated in vitro with 10µM of RO5459072 or DMSO, and lysed for detection of Lip10 accumulation by Western blotting. The antibody raised against Lip10 neoepitope was used as primary antibody. COX IV protein detection was used as a loading control.

**
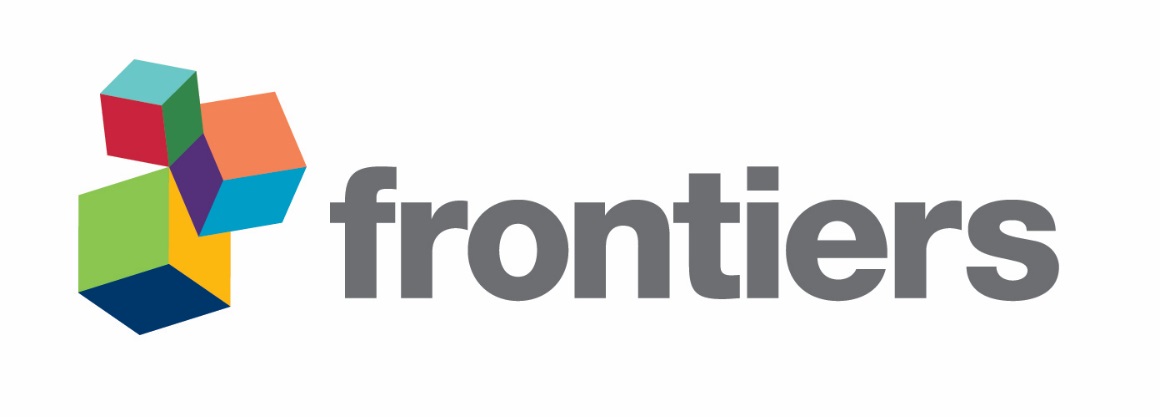
**

**
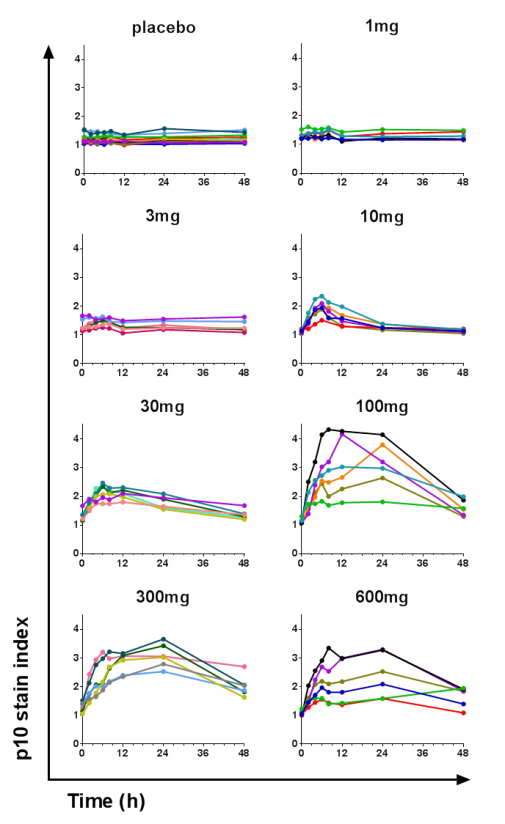
**

**Supplementary Figure 2.** Lip10 accumulation in B cells of healthy individuals administered a single oral dose of CatS inhibitor RO5459072. Healthy volunteers enrolled in a single ascending dose clinical study of RO5459072 were given a single oral dose of the CatS inhibitor ranging from 1 to 600mg or a placebo control. Data represent p10 stain index in circulating B cells of individual healthy volunteers, grouped per graph by oral dose received.


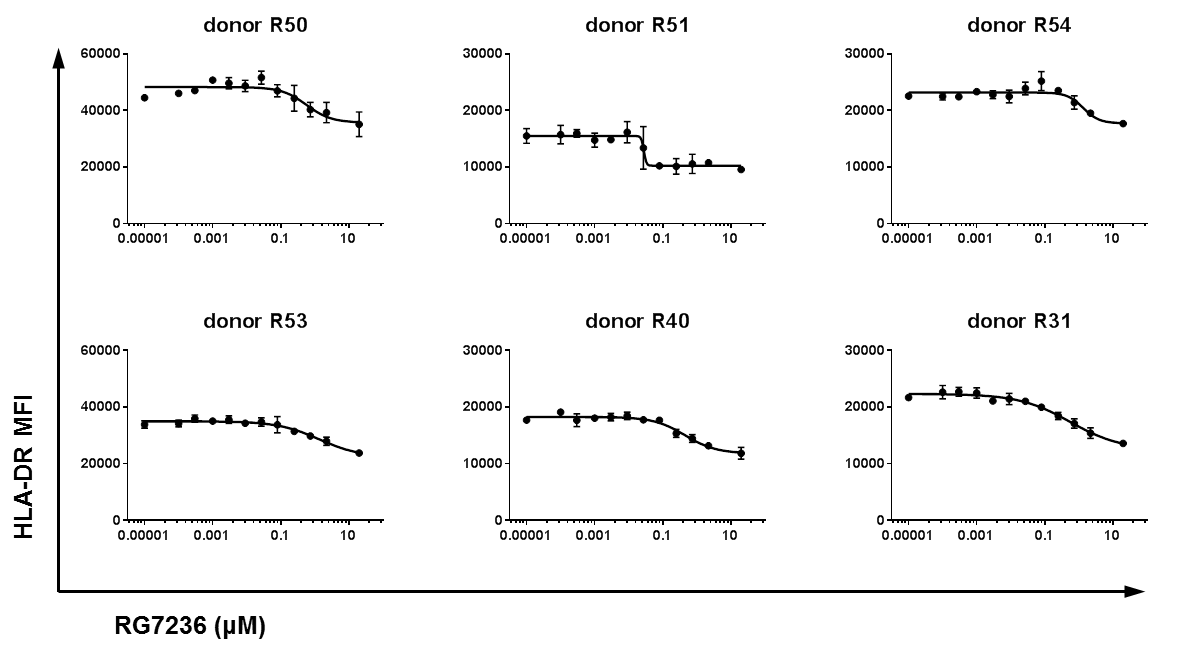


**Supplementary Figure 3.** Incubation of PBMC with CatS inhibitor RG7236 led to a reduction in MHC II surface expression by B cells. Detection of HLA-DR surface expression by flow cytometry in B cells from 6 healthy volunteers after in vitro incubation of PBMC with a serial titration of RG7236. Curve fit was determined by non-linear regression using a variable slope model with GraphPad Prism software.

## Supplementary Tables

| **Target epitope** | | 116 | | | 117 | | | 118 | | | 120 | | |
| --- | --- | --- | --- | --- | --- | --- | --- | --- | --- | --- | --- | --- | --- |
| **Clone** | | 1C4 | 1E4 | 3B7 | 6H9 | 7B8 | 13C4 | 3D6 | 3G10 | 2C11 | 5C9 | 7G6 | 8C2 |
| **direct** | 116 | +++ | +++ | +++ | - | - | - | - | - | - | - | - | - |
|  | 117 | +++ | +++ | +++ | + | + | + | - | - | - | - | - | - |
|  | 118 | + | + | + | - | - | - | +++ | +++ | +++ | ++ | ++ | ++ |
|  | 120 | - | - | - | - | - | - | - | - | - | +++ | +++ | +++ |
| **saandwich** | 116 | - | - | - | - | - | - | - | - | - | ++ | ++ | ++ |
|  | 117 | +++ | ++ | ++ | ++ | ++ | ++ | - | - | - | - | - | - |
|  | 118 | +++ | ++ | ++ | - | - | - | - | - | - | - | - | - |
|  | 120 | - | - | - | - | - | - | - | - | - | +++ | +++ | +++ |

**Supplementary Table 1. Monoclonal antibody specificity for Lip10 neoepitopes.** Specificity of monoclonal antibodies raised in rabbits following immunization with peptides corresponding to alternatively cleaved C-terminal ends of Lip10 (neoepitope), determined by ELISA. For the direct ELISA streptavidin plates were directly coated with the corresponding biotinylated peptides. For the sandwich ELISA corresponding fusion peptides consisting of an N-terminal epitope of Lip10 and the various C-terminal ends were used. The fusion peptides were captured with an antibody specific for the N-terminal portion (PIN.1). Data represent intensity of signal detected, ranging from no signal (-) to bright signal (+++).
